# Physical Activity Status and Adiposity-Related Morphological Risk in Children: Differences in Anthropometric, Body Composition, and Somatotype Indicators

**DOI:** 10.3390/healthcare14152402

**Published:** 2026-08-05

**Authors:** Eduardo Guzmán-Muñoz, Yeny Concha-Cisternas, Exal Garcia-Carrillo, Igor Cigarroa, Felipe Montalva-Valenzuela, Iván Molina-Marquez, Rodrigo Yáñez-Sepúlveda

**Affiliations:** 1 Escuela de Kinesiología, Facultad de Salud, Universidad Santo Tomás, Talca 3460000, Chile; yenyconchaci@santotomas.cl; 2 Escuela de Pedagogía en Educación Física, Facultad de Educación, Universidad Autónoma de Chile, Talca 3460000, Chile; 3 Vicerrectoría de Investigación e Innovación, Universidad Arturo Prat, Iquique 1100000, Chile; 4 Department of Physical Activity Sciences, Faculty of Education Sciences, Universidad Católica del Maule, Talca 3480112, Chile; 5 Department of Physical Activity Sciences, Universidad de Los Lagos, Osorno 5290000, Chile; 6 Escuela de Kinesiología y Terapia Ocupacional, Facultad de Ciencias de la Salud, Universidad Católica Silva Henriquez, Santiago 8320000, Chile; icigarroac@ucsh.cl; 7 Escuela de Entrenador en Actividad Física y Deporte, Facultad de Ciencias Humanas, Universidad Bernardo O’Higgins, Santiago 8370040, Chile; felipe.montalva@ubo.cl; 8 Pedagogía en Educación Física, Facultad de Educación, Universidad Adventista de Chile, Chillán 3780000, Chile; ivanmolina@unach.cl; 9 Programa de Doctorado en Ciencias de la Actividad Física, Universidad Católica del Maule, Talca 3460000, Chile; 10 Facultad de Educación y Humanidades, Escuela de Ciencias del Deporte, Universidad Andres Bello, Viña del Mar 2200055, Chile; rodrigo.yanez.s@unab.cl; 11 School of Medicine, Universidad Espíritu Santo, Samborondón 092301, Ecuador

**Keywords:** pediatric obesity, waist circumference, conicity index, fat mass index, endomorphy, sedentary behavior, school health

## Abstract

**Background/Objectives:** Physical inactivity represents one of the most critical public health challenges of recent decades, particularly in pediatric populations. Therefore, this study aimed to evaluate differences in adiposity-related morphological risk indicators between physically active and inactive children by simultaneously integrating anthropometric measures, five-component body composition, and somatotype analysis. **Methods:** This cross-sectional study included 126 children aged 6–9 years (56 girls and 70 boys). Physical activity status was determined using the Physical Activity Questionnaire for Older Children. Anthropometric indicators included BMI, waist circumference, waist-to-hip ratio, waist-to-height ratio, and conicity index. Adipose and muscle mass, fat mass index, and adipose-to-muscle ratio were estimated using Kerr’s five-component model, and somatotype was assessed using the Heath-Carter method. **Results:** Inactive children showed higher body mass index (BMI), waist circumference, waist-to-hip ratio, waist-to-height ratio, conicity index, adipose mass, fat mass index, sum of skinfolds, and endomorphy, together with lower ectomorphy, than active children (all *p* < 0.05). Sex-stratified analyses showed that these differences were broader and generally larger among boys. Age-adjusted interaction models also indicated more pronounced active–inactive differences in boys for several central and total adiposity indicators. **Conclusions:** Physical inactivity was associated with a less favorable adiposity-related morphological profile in children aged 6–9 years, particularly among boys. These findings support complementing BMI with central adiposity indicators, body composition measures, and somatotype analysis in pediatric and school-based assessments, potentially supporting earlier identification of children with unfavorable morphological profiles.

## 1. Introduction

Physical inactivity has become one of the most critical public health challenges of the 21st century, particularly among the pediatric population [1,2]. The reduction in daily energy expenditure, together with the increase in sedentary behaviors, has contributed to an unfavorable cardiometabolic profile from early stages of life [3,4]. The prepubertal period represents a critical window for morphological and metabolic development; therefore, understanding how physical activity status is associated with body composition and adiposity distribution during this stage is essential for early prevention strategies [5]. Childhood overweight and obesity represent a growing global public health challenge, with consequences that may extend from early life into adolescence and adulthood [3,4]. Current international guidance emphasizes the importance of promoting regular physical activity and reducing sedentary behavior as part of population-level strategies to prevent excess adiposity and associated cardiometabolic risk in children [6,7]. Accordingly, identifying unfavorable adiposity-related profiles before puberty may support earlier and more targeted preventive actions [5,6].

Traditionally, epidemiological studies have relied on body mass index (BMI) to classify nutritional status and estimate obesity-related health risk [8]. However, BMI has an important methodological limitation: it does not distinguish between fat mass and fat-free mass, nor does it provide information about the anatomical distribution of adipose tissue [8,9]. Consequently, children classified within normal weight categories may still present unfavorable body composition profiles, including relatively higher adiposity and lower muscle mass, which may be overlooked when BMI is used as the only screening indicator [10,11]. Adiposity-related morphological risk refers to an unfavorable combination of body size, central and subcutaneous adiposity, body composition, and somatotype characteristics. This construct does not constitute a clinical diagnosis or a direct measure of cardiometabolic disease. From a public health perspective, identifying these unfavorable morphological profiles during childhood is important because excess central and total adiposity may develop before conventional BMI-based classifications adequately reflect the underlying body composition pattern. Therefore, a multidimensional assessment that integrates body size, adiposity distribution, tissue composition, and overall body shape may provide a more informative characterization of early adiposity-related morphological risk.

Current evidence suggests that the distribution of adipose tissue may be more informative than total body mass for identifying early cardiometabolic risk [12,13]. In particular, central adiposity, characterized by greater adipose tissue accumulation in the trunk and abdominal region, has been associated with metabolic alterations such as insulin resistance, low-grade inflammation, and increased cardiovascular risk [13]. In this context, anthropometric indices such as waist-to-height ratio (WHtR) and the conicity index have been proposed as practical, non-invasive tools for identifying adiposity-related cardiometabolic risk, complementing the information provided by traditional weight-for-height indicators [14].

Furthermore, a more comprehensive assessment of the morphological impact of physical inactivity requires the integration of body composition and somatotype indicators. Kerr’s five-component body composition model provides a detailed anthropometric approach by fractionating body mass into adipose, muscle, bone, residual, and skin tissue compartments [15]. This allows the estimation of clinically relevant indicators such as the adipose-to-muscle ratio (AMR), which reflects the balance between adipose tissue accumulation and muscle mass, a key tissue involved in glucose uptake and metabolic regulation. The Heath-Carter somatotype method was included as an exploratory descriptor of body shape through endomorphy, mesomorphy, and ectomorphy. Because these components partly reflect adiposity, musculoskeletal robustness, and body linearity, they were interpreted as complementary rather than independent morphological indicators [16]. Together with central adiposity indicators, these approaches provide complementary information on body size, adiposity distribution, tissue composition, and morphological phenotype, allowing a broader characterization than BMI alone.

Although the benefits of physical activity during childhood are well established, few studies have simultaneously examined physical activity status in relation to central adiposity indicators, five-component body composition, adipose-to-muscle balance, and somatotype in children aged 6–9 years. Most previous studies have relied on BMI or isolated anthropometric measures, which may not fully characterize adiposity distribution, the relative contribution of adipose and muscle tissues, or the overall morphological phenotype. Moreover, it remains unclear whether active–inactive differences in these complementary morphological indicators vary by sex during the prepubertal period, when biological, maturational, and behavioral differences may begin to influence body composition and adiposity distribution.

Therefore, the primary objective of this study was to compare anthropometric, body composition, and somatotype indicators between physically active and inactive children aged 6–9 years. The secondary, exploratory objective was to determine whether the magnitude of these differences varied according to sex. Such evidence may inform feasible pediatric and school-based assessment strategies that complement BMI and support the early identification of children with less favorable morphological profiles.

## 2. Materials and Methods

### 2.1. Study Design

This was a quantitative, descriptive-comparative study with an observational cross-sectional design [17]. The study was conducted in two public primary schools in Talca, a city located in central Chile. The school setting was selected because it provided access to children within the target age range and allowed anthropometric and questionnaire-based assessments to be conducted under standardized conditions within their usual educational environment. The study protocol was approved by the Ethics Committee of Universidad Santo Tomás before data collection (protocol code 114-16; approval date: 30 January 2021). The study was conducted in accordance with the Declaration of Helsinki.

### 2.2. Participants

The study sample consisted of 126 children aged 6 to 9 years (56 girls and 70 boys), recruited from two public schools in the city of Talca, Chile. Participants were selected using a non-probabilistic convenience sampling strategy, according to the availability of eligible children and the voluntary participation of their parents or legal guardians. Written informed consent was obtained from parents or legal guardians, and child assent was requested before the assessments.

Children were eligible for inclusion if they met the following criteria: age between 6 and 9 years; enrollment in a school setting at the time of evaluation; apparent healthy status according to parent or guardian report; ability to complete the anthropometric, body composition, somatotype, and PAQ-C assessments; and availability of signed informed consent from a parent or legal guardian, together with child assent.

Children were excluded if they presented any diagnosed endocrine, metabolic, cardiovascular, neurological, genetic, or musculoskeletal condition that could affect growth, body composition, physical activity participation, or the interpretation of anthropometric indicators. Children were also excluded if they had an acute illness or recent injury that limited normal movement or participation in physical activity; were receiving pharmacological treatment known to affect growth, body weight, appetite, or body composition, such as systemic corticosteroids or growth-related medication; had incomplete anthropometric, body composition, somatotype, or PAQ-C data; or were unable to follow the assessment procedures.

Based on a previous study comparing physically active and inactive children, the observed effect size for waist circumference was large (Cohen’s d = 0.89) [18]. Therefore, the sample size was estimated using waist circumference as the reference central adiposity indicator. This calculation was not intended to ensure adequate statistical power for all secondary anthropometric, body composition, and somatotype outcomes. Considering a two-tailed alpha level of 0.05, a statistical power of 95%, an allocation ratio of 1:1, and an expected effect size of Cohen’s ES = 0.89, a minimum of 34 participants per group was required, resulting in an estimated total sample size of 68 children. To account for potential incomplete data, an additional 10% was considered, increasing the target sample to approximately 75 children.

### 2.3. Anthropometric Profile and Body Composition

Anthropometric assessments were performed according to the recommendations of the International Society for the Advancement of Kinanthropometry (ISAK). ISAK provides international standards for anthropometric assessment and promotes quality control through the technical error-of-measurement framework. All measurements were performed by a single evaluator trained in standardized ISAK procedures under standardized conditions. Participants were assessed in a school setting, wearing light clothing and without shoes. Before the assessment, children were instructed to stand in the anatomical position, with their bodies relaxed, and to follow the evaluator’s instructions.

Age was recorded in years. Body mass was measured in kilograms using a digital scale (Seca, Hamburg, Germany; precision 0.1 kg), and standing height was measured in centimeters using a stadiometer (Seca, Hamburg, Germany; precision 0.1 cm). The BMI was calculated by dividing body mass in kilograms by standing height squared in meters and expressed as kg/m^2^.

Waist circumference was measured at the minimum waist using a non-elastic anthropometric tape (Sanny, São Paulo, Brazil; precision 0.1 cm), with the participant standing upright, abdomen relaxed, arms positioned alongside the body, feet together, and the measurement taken at the end of a normal expiration. Hip circumference was measured at the maximum gluteal protuberance using the same tape, with the participant standing upright and feet together. These measurements were used to calculate the WHtR, obtained by dividing waist circumference by height, both expressed in the same unit of measurement, and the waist-to-hip ratio (WHR), obtained by dividing waist circumference by hip circumference.

The conicity index was calculated as an anthropometric indicator of central adiposity using waist circumference, body mass, and height. This index allows the evaluation of the degree to which body shape approximates a double-cone morphology, which is associated with greater abdominal adipose tissue accumulation [19]. Therefore, waist circumference, WHtR, WHR, and the conicity index were considered central adiposity-related anthropometric indicators.

In addition to basic anthropometry, a complete kinanthropometric profile was obtained. Bone breadths were measured using anthropometers (Rosscraft, Vancouver, BC, Canada; precision 0.1 mm), girths were measured with a non-elastic anthropometric tape (Sanny, São Paulo, Brazil; precision 0.1 cm), and skinfolds were measured using a skinfold caliper (Harpenden, Hertfordshire, UK; precision 0.2 mm). The anthropometric profile included six bone breadths: biacromial, transverse thorax, anteroposterior thorax, bicrestal, biepicondylar humerus, and biepicondylar femur. Ten girths were assessed: head, relaxed arm, flexed arm in tension, maximum forearm, mesosternal thorax, minimum waist, maximum hip, maximal thigh, medial thigh, and maximal calf. Six skinfolds were measured: triceps, subscapular, supraspinal, abdominal, medial thigh, and maximal calf. The sum of six peripheral skinfolds was calculated as an indicator of subcutaneous adiposity. Higher values were interpreted as reflecting greater subcutaneous adipose tissue accumulation.

Body composition was estimated using Kerr’s five-component anthropometric model, which fractionates total body mass into adipose, muscle, bone, residual, and skin masses [15]. The original Kerr model was developed for males and females aged 6–77 years, thereby encompassing the age range of the participants included in the present study [15]. Nevertheless, the resulting body composition values were interpreted as model-derived anthropometric estimates rather than direct measurements of tissue mass. In the present study, adipose mass and muscle mass were retained as the main body composition indicators because they allow characterization of the balance between adiposity and muscularity. Adipose mass was expressed in kilograms and interpreted as the absolute amount of adipose tissue. Muscle mass was also expressed in kilograms and interpreted as the absolute amount of skeletal muscle tissue estimated from anthropometric variables.

From these values, the AMR was calculated by dividing adipose mass by muscle mass. This ratio was used as an indicator of the proportional balance between adipose tissue and muscle tissue, where higher values indicate a less favorable morphological profile characterized by greater adiposity relative to muscularity. In addition, the fat mass index (FMI) was calculated by dividing adipose mass in kilograms by standing height squared in meters and expressed as kg/m^2^. FMI was used to normalize adipose mass by body size and to provide a height-adjusted adiposity indicator.

Somatotype was calculated according to the Heath-Carter anthropometric method, which quantifies body shape and composition through three numerical components: endomorphy, mesomorphy, and ectomorphy. Endomorphy represents relative adiposity, mesomorphy represents musculoskeletal robustness, and ectomorphy represents relative linearity [16]. These three components were used to characterize the morphological phenotype of the participants and to compare somatotype patterns according to physical activity status and sex.

Thus, the complete set of anthropometric and morphological variables included age, body mass, standing height, BMI, minimum waist circumference, WHtR, conicity index, WHR, adipose mass, muscle mass, AMR, FMI, sum of six skinfolds, endomorphy, mesomorphy, and ectomorphy. These measures were selected because they represent complementary dimensions of adiposity-related morphology. BMI was included as the conventional indicator of body size relative to height, whereas waist circumference, WHR, WHtR, and the conicity index were used to characterize central adiposity and adiposity distribution. Kerr’s five-component model was applied to estimate adipose and muscle mass and to examine the proportional balance between these tissues. Finally, the Heath-Carter somatotype method was included to provide an integrated description of relative adiposity, musculoskeletal robustness, and body linearity. The combined use of these indicators allowed a broader assessment of morphological differences associated with physical activity status than would have been possible using BMI alone.

### 2.4. Physical Activity Status

Physical activity status was assessed using the Physical Activity Questionnaire for Older Children (PAQ-C). The PAQ-C evaluates general physical activity performed during the previous seven days and provides a summary score based on nine items rated on a five-point scale, where 1 indicates low physical activity and 5 indicates high physical activity [20,21]. The items assess physical activity performed during leisure time, physical education classes, recess, lunchtime, after school, evenings, and weekends. A tenth item, which is not included in the final score, identifies unusual circumstances during the previous week that may have affected the child’s habitual physical activity. The final PAQ-C score was calculated as the mean of the nine scored items.

The PAQ-C was originally developed primarily for older school-aged children and has most commonly been used in children aged approximately 8–14 years [20,21]. Because the present study included children aged 6–9 years, the questionnaire was completed by the children with assistance from their parents or legal guardians when necessary. Parents or guardians helped younger participants understand the questions and recall the physical activities performed during the previous seven days, without selecting or modifying their responses.

PAQ-C scores were used to establish an operational classification of physical activity status. A cut-off score of 2.73, previously proposed by Benítez-Porres et al. in relation to the achievement of more than 60 min of moderate-to-vigorous physical activity per day, was used for this purpose [22]. Children with PAQ-C scores ≥ 2.73 were classified as physically active, whereas those with scores < 2.73 were classified as physically inactive [22]. However, because the discriminatory performance of this threshold was limited and non-significant in the source study, the categorization was used for exploratory analytical purposes and should not be interpreted as a validated diagnostic classification of physical activity status.

### 2.5. Statistical Analysis

Statistical analyses were performed using GraphPad Prism software, version 8.0 (GraphPad Software, San Diego, CA, USA). Data normality was assessed using the Shapiro–Wilk test, and homoscedasticity was verified with Levene’s test. Descriptive statistics were presented as means and standard deviations (SD). For the initial analysis, differences in anthropometric, body composition, and somatotype variables between active and inactive children were evaluated using the independent-samples *t*-test for the total sample.

To explore sex-specific patterns, the sample was stratified, and independent-samples *t*-tests were performed separately for boys and girls. The magnitude of the differences was estimated using Cohen’s d, calculated as the difference between the group means divided by the pooled standard deviation of the two groups. Absolute Cohen’s d values were reported and interpreted as trivial (<0.20), small (0.20–0.49), moderate (0.50–0.79), and large (≥0.80). Furthermore, to determine whether differences in morphological indicators between physically active and inactive children varied by sex, separate multiple linear regression models were fitted for each continuous morphological outcome using the multiple regression procedure available in GraphPad Prism 8.0. Each model included physical activity status, sex, age as a continuous covariate, and the physical activity status × sex interaction term. Physical activity status was coded as 0 = inactive and 1 = active, whereas sex was coded as 0 = girls and 1 = boys. Interaction effects were reported as unstandardized β coefficients with 95% confidence intervals. The interaction coefficient represented the difference between the active–inactive contrast in boys and the corresponding active–inactive contrast in girls. Under this coding, a negative interaction coefficient indicated that the active–inactive contrast was more negative in boys than in girls. Given the exploratory nature of the analyses and the inclusion of several closely related morphological indicators, *p*-values were not adjusted for multiple comparisons. Findings were interpreted together with Cohen’s d effect sizes for group comparisons and 95% confidence intervals for the interaction models. For all statistical tests, a two-tailed *p*-value <0.05 was considered statistically significant.

## 3. Results

A total of 126 children were included in the study, of whom 56 were girls and 70 were boys. According to physical activity status, 66 children were classified as physically active and 60 as physically inactive. Girls had a mean age of 7.64 ± 1.13 years, body weight of 33.91 ± 10.31 kg, and height of 129.85 ± 9.73 cm. Boys had a mean age of 7.91 ± 1.12 years, body weight of 34.35 ± 9.72 kg, and height of 130.61 ± 9.41 cm.

In the total sample, physically inactive children showed significantly higher body weight (*p* = 0.020; ES = 0.42), BMI (*p* = 0.004; ES = 0.52), waist circumference (*p* = 0.001; ES = 0.63), WHR (*p* < 0.001; ES = 0.68), WHtR (*p* < 0.001; ES = 0.71), conicity index (*p* < 0.001; ES = 0.84), adipose mass (*p* = 0.044; ES = 0.36), FMI (*p* = 0.025; ES = 0.41), sum of skinfolds (*p* = 0.017; ES = 0.43), and endomorphy (*p* = 0.003; ES = 0.53) compared with physically active children. In contrast, physically inactive children showed significantly lower ectomorphy (*p* = 0.004; ES = 0.52). No significant differences were observed for age, height, muscle mass, AMR, or mesomorphy (Table 1).

When analyses were stratified by sex, inactive girls presented significantly higher BMI (*p* = 0.008; ES = 0.76), waist circumference (*p* = 0.021; ES = 0.73), WHR (*p* = 0.036; ES = 0.46), WHtR (*p* = 0.040; ES = 0.33), conicity index (*p* = 0.047; ES = 0.46), and endomorphy (*p* = 0.037; ES = 0.42) than active girls (Table 2). No significant differences were found for age, weight, height, adipose mass, muscle mass, AMR, FMI, sum of skinfolds, mesomorphy, or ectomorphy.

Among boys, physically inactive participants showed significantly higher weight (*p* = 0.006; ES = 0.68), BMI (*p* = 0.010; ES = 0.77), waist circumference (*p* < 0.001; ES = 0.91), WHR (*p* < 0.001; ES = 0.84), WHtR (*p* < 0.001; ES = 0.88), conicity index (*p* < 0.001; ES = 1.12), adipose mass (*p* = 0.010; ES = 0.65), FMI (*p* = 0.015; ES = 0.66), sum of skinfolds (*p* = 0.003; ES = 0.67), and endomorphy (*p* = 0.012; ES = 0.72) compared with active boys (Table 3). No significant differences were observed for age, height, muscle mass, AMR, mesomorphy, or ectomorphy.

**Figure 1:**
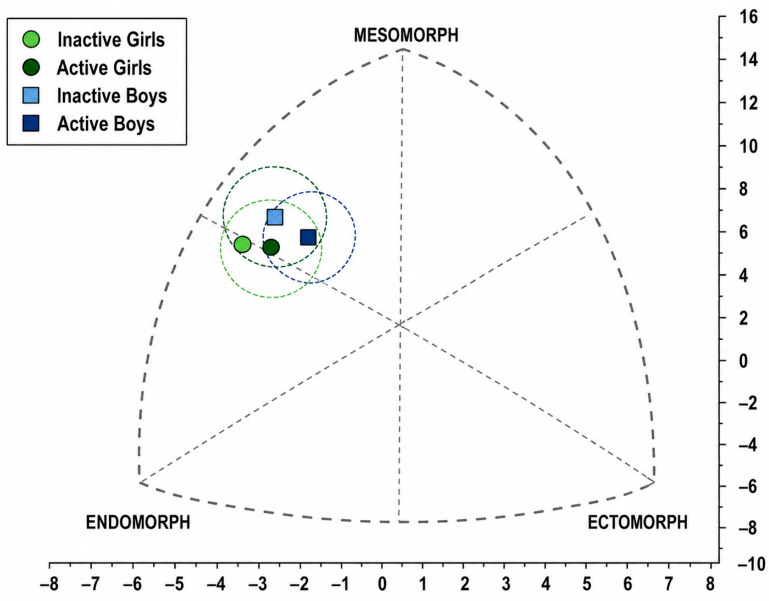
Mean somatotype profiles according to sex and physical activity status. Each point represents the mean somatotype coordinates of one group: active girls, inactive girls, active boys, and inactive boys. Somatotype coordinates were calculated from the three Heath–Carter components—endomorphy, mesomorphy, and ectomorphy—and plotted on the somatochart. The circular symbols represent group mean coordinates only and do not indicate standard deviations, confidence intervals, confidence ellipses, or individual-level dispersion.

To complement the numerical comparisons, the mean somatotype profile of each sex and physical activity group was descriptively represented using a somatochart. The visual analysis showed that inactive girls were positioned further toward the endomorphic region, whereas active girls showed a slightly less endomorphic profile. A similar pattern was observed among boys, with inactive boys showing a more endomorphic location than active boys. However, both groups of boys were positioned higher on the somatochart, reflecting a greater mesomorphic component compared with girls. Overall, the somatochart provides a descriptive visualization of the mean group profiles and is consistent with the higher endomorphy observed among physically inactive children. However, it was not intended to evaluate individual dispersion or to function as an independent discriminatory tool (Figure 1).

After adjustment for age, significant sex × physical activity interactions were observed for body weight (β = −3.13; 95% CI: −6.24 to −0.02; *p* = 0.048), height (β = −5.47; 95% CI: −10.45 to −0.49; *p* = 0.032), waist circumference (β = −2.47; 95% CI: −4.84 to −0.10; *p* = 0.041), WHR (β = −0.03; 95% CI: −0.05 to −0.01; *p* = 0.018), WHtR (β = −0.02; 95% CI: −0.04 to −0.002; *p* = 0.026), conicity index (β = −0.04; 95% CI: −0.07 to −0.01; *p* = 0.006), adipose mass (β = −0.05; 95% CI: −0.09 to −0.01; *p* = 0.021), FMI (β = −0.02; 95% CI: −0.04 to −0.001; *p* = 0.047), and sum of skinfolds (β = −7.51; 95% CI: −14.63 to −0.39; *p* = 0.039) (Table 4). No significant interactions were observed for age, BMI, muscle mass, AMR, endomorphy, mesomorphy, or ectomorphy. Overall, the significant negative interaction coefficients indicate that the differences between physically active and inactive children were more pronounced in boys than in girls, particularly for indicators of central and total adiposity.

## 4. Discussion

The present study examined whether physical activity status was associated with adiposity-related morphological risk in children aged 6–9 years, considering anthropometric, body composition, and somatotype indicators. The main findings indicate that physically inactive children showed a less favorable adiposity-related morphological profile, characterized by higher body weight, BMI, waist circumference, WHR, WHtR, conicity index, adipose mass, FMI, sum of skinfolds, and endomorphy, together with lower ectomorphy, compared with their physically active peers. These differences were particularly evident for central adiposity indicators, suggesting that physical inactivity in early childhood was associated not only with greater body size, but also with a more unfavorable pattern of adiposity distribution. Importantly, sex-stratified analyses and age-adjusted interaction models suggested that the differences between physically active and inactive children tended to be more pronounced in boys, especially for waist circumference, WHR, WHtR, conicity index, adipose mass, FMI, and sum of skinfolds. These findings are consistent with current evidence indicating that physical activity and movement behaviors during childhood are associated with adiposity and broader health indicators, including cardiometabolic health [23,24,25].

A central finding of this study was that physically inactive children presented higher values in several adiposity-related indicators. This is relevant because childhood physical inactivity has been recognized as a major public health concern, and current international guidelines recommend that children and adolescents accumulate an average of at least 60 min per day of moderate-to-vigorous physical activity [7]. The 2020 WHO guidelines highlight that higher physical activity and lower sedentary behavior are associated with more favorable adiposity and cardiometabolic profiles in children and adolescents [6]. In line with this evidence, recent systematic reviews based on 24-h movement behavior frameworks have shown that adherence to movement guidelines, including sufficient physical activity, limited sedentary time, and adequate sleep, is generally associated with lower overweight and obesity indicators and more favorable health outcomes in pediatric populations [26]. Therefore, the present findings reinforce the importance of identifying unfavorable morphological profiles associated with low physical activity before puberty, when preventive strategies may still have greater potential to modify future health trajectories.

A relevant aspect of the present study is that differences between physically active and inactive children were more evident in central adiposity indicators than in total body composition variables. In the total sample, physically inactive children showed higher waist circumference, WHR, WHtR, conicity index, adipose mass, FMI, and sum of six skinfolds, whereas muscle mass and AMR did not differ significantly. This pattern suggests that physical inactivity may be more strongly related to the distribution of adiposity than to total adipose tissue alone. This interpretation is supported by evidence showing that abdominal and central adiposity are closely linked to cardiometabolic risk in children and adolescents. In particular, WHtR has been proposed as a practical screening indicator because it incorporates waist circumference relative to height and may better reflect central adiposity accumulation than BMI alone. A meta-analysis reported that WHtR has useful performance for identifying cardiometabolic risk clustering in children, supporting its use as a complementary anthropometric indicator in pediatric risk assessment [27]. Similarly, evidence from pediatric populations with obesity indicates that abdominal adiposity and total adiposity are relevant predictors of cardiometabolic health, reinforcing the clinical relevance of central adiposity indicators [28].

The higher conicity index observed in inactive children also deserves attention. Unlike BMI, the conicity index incorporates waist circumference, body mass, and height, providing information about the extent to which body shape approximates a more abdominally concentrated pattern of adiposity. In the present study, the conicity index showed one of the largest effect sizes, particularly in boys, suggesting that this indicator may be sensitive to physical activity-related morphological differences during the prepubertal period. This finding is clinically relevant because central adiposity may be present even when BMI does not fully capture the underlying adiposity-related risk. It has been shown that BMI-based obesity classification may fail to identify children and adolescents with increased adiposity and elevated cardiometabolic risk, supporting the need to complement BMI with more specific adiposity indicators [11]. Thus, the combined interpretation of BMI, waist circumference, WHtR, WHR, and conicity index may provide a more complete picture of early morphological risk than BMI alone.

Muscle mass was higher among physically inactive children, although the difference did not reach statistical significance (*p* = 0.068; Cohen’s d = 0.43), whereas AMR showed no significant difference between groups. These findings should not be interpreted as evidence that physical inactivity has no relationship with the muscular component. Rather, it may indicate that, in children, the early morphological expression of inactivity is more detectable through adiposity distribution than through absolute muscle mass. This interpretation is plausible because skeletal muscle development during childhood is strongly influenced by growth, maturation, sex, and body size. The evidence suggests that adiposity-related risk in pediatric populations may be underestimated when assessment is limited to body weight or BMI, highlighting the need to integrate body composition and adiposity distribution indicators [11,28]. Therefore, the lack of significant differences in estimated muscle mass does not diminish the relevance of the adiposity-related findings; instead, it suggests that the earliest detectable differences in this sample were mainly expressed through central and subcutaneous adiposity markers.

The numerical somatotype components showed higher endomorphy among physically inactive children in the total sample and in the sex-stratified analyses. This pattern was consistent with the higher adipose mass and central adiposity indicators observed particularly in the total sample and among boys. In contrast, mesomorphy did not differ significantly according to physical activity status, and differences in ectomorphy were not consistent across the sex-stratified analyses. Somatotype has been described as being influenced by both genetic and environmental factors and as being associated with physical fitness-related characteristics, supporting its use as a complementary morphological descriptor during growth [29]. Therefore, in the present analysis, somatotype was interpreted as an exploratory and complementary descriptor. The somatochart was retained only as a descriptive visualization of the mean group profiles and not as an independent discriminatory analysis.

Sex-specific patterns were also observed in the association between physical activity status and adiposity-related morphological indicators. In girls, physical inactivity was associated with higher BMI, waist circumference, WHR, WHtR, conicity index, and endomorphy, whereas no significant differences were observed in adipose mass, FMI, or the sum of six skinfolds. Among boys, physically inactive participants showed higher values not only in central adiposity indicators but also in body weight, adipose mass, FMI, the sum of six skinfolds, and endomorphy. Moreover, the interaction models indicated that the differences between physically active and inactive children were significantly greater in boys for several adiposity-related variables. These findings are consistent with international evidence showing that the associations among physical activity, sedentary behavior, and adiposity may differ according to sex, behavioral context, and activity intensity [30,31]. The present study extends this evidence by documenting these patterns in Chilean children aged 6–9 years using complementary anthropometric, body composition, and somatotype indicators.

Several explanations may account for this sex-specific pattern. First, boys and girls may differ in the type, intensity, and context of physical activity, even when classified using the same questionnaire-based cut-off. Second, differences in growth tempo and early maturation-related processes may influence body composition and adiposity distribution. Third, physical activity in boys may involve greater variability in vigorous play, sport participation, and energy expenditure, contrasting physically active and inactive boys more pronouncedly. This interpretation is consistent with studies showing that the characteristics of moderate-to-vigorous physical activity, not only its total duration, may be relevant for adiposity outcomes in children [31]. However, because the present study did not assess pubertal stage, dietary intake, sedentary time, or objectively measured physical activity intensity, these explanations should be interpreted cautiously. The findings should also be interpreted within the context of children attending public schools in Talca, central Chile. Opportunities for structured physical activity, access to recreational spaces, family resources, school characteristics, and local sociocultural patterns may influence both physical activity behaviors and adiposity-related morphology. However, these contextual factors were not directly assessed and should be examined in future multicenter studies involving children from different educational, socioeconomic, and geographic settings.

In this sample, the largest and most consistent differences between physically active and inactive children were observed in waist circumference, WHR, WHtR, and the conicity index. This finding is consistent with evidence showing that BMI-based classifications may overlook children with increased adiposity and cardiometabolic risk [11], as well as with meta-analytic evidence supporting WHtR as a useful complementary screening indicator in pediatric populations [27]. Therefore, central adiposity indicators provided relevant information beyond BMI when characterizing the adiposity-related morphological differences associated with physical activity status.

Beyond its morphological contribution, the present study has practical implications for school health, pediatric rehabilitation, and early prevention strategies. These findings suggest that anthropometric surveillance in school and pediatric settings should not be limited to body weight or BMI, but should also include simple and feasible indicators of central adiposity, such as waist circumference and waist-to-height ratio. This is particularly relevant because physical inactivity was associated with measurable morphological differences even in children aged 6 to 9 years, a period that precedes adolescence, when physical activity often declines and adiposity patterns may become more difficult to reverse. For physical education teachers, school health teams, pediatric physiotherapists, and other health professionals, the combined interpretation of central adiposity, body composition, and somatotype indicators may support earlier identification of children with less favorable morphological profiles. This approach may also guide more individualized counseling, timely referral, and the design of interventions focused not only on body weight control, but also on reducing central adiposity accumulation and improving movement behaviors. Finally, the stronger differences observed among boys suggest that sex-specific patterns should be considered when designing school- and community-based physical activity programs. At the policy level, these findings support strengthening school-based physical activity promotion and the use of feasible central adiposity indicators as complements to BMI in pediatric and school health surveillance; however, longitudinal evidence is needed before translating the observed sex-specific patterns into differentiated screening recommendations.

Several limitations should be considered when interpreting these findings. First, the cross-sectional design precludes causal inference and does not allow us to determine whether lower physical activity contributed to a less favorable adiposity-related morphological profile or whether children with greater adiposity were less likely to engage in physical activity. Second, participants were recruited through convenience sampling from two public schools in a single Chilean city. Although the final sample exceeded the minimum sample size estimated a priori, the relatively modest sample and geographically restricted recruitment may limit the generalizability of the findings to children from other educational, socioeconomic, cultural, or geographic contexts. Third, physical activity status was assessed using the PAQ-C rather than objective methods such as accelerometry. Although this questionnaire is practical for school-based research, responses may be affected by recall, comprehension, and reporting bias, particularly in younger children, even when parental assistance is provided. Fourth, potentially relevant variables, including dietary intake, socioeconomic status, sedentary behavior, sleep duration, screen time, environmental opportunities for physical activity, and pubertal maturation, were not assessed. Therefore, their potential influence on the observed associations could not be determined. In addition, because regional and cultural factors were not directly measured, their contribution to physical activity patterns and adiposity-related morphology could not be evaluated. Fifth, the sex-stratified analyses involved smaller subgroup sample sizes and should therefore be interpreted as exploratory. Finally, the analyses involved multiple comparisons across several related morphological indicators without formal adjustment for multiplicity. Consequently, the possibility of type I error cannot be excluded, and individual statistically significant findings should be interpreted as exploratory and considered in conjunction with their effect sizes and the overall consistency of the observed pattern.

Despite these limitations, the study has several strengths. First, it used a multidimensional assessment that extended beyond BMI by integrating central adiposity indicators, Kerr’s five-component body composition model, skinfold measurements, and Heath-Carter somatotype analysis. This approach allowed physical activity-related differences to be examined across complementary dimensions of body size, adiposity distribution, tissue composition, and overall morphological phenotype. Second, anthropometric assessments were conducted by trained evaluators under standardized ISAK-based procedures, while physical activity and somatotype were assessed using established instruments and methods. Third, the inclusion of sex-stratified comparisons and age-adjusted sex × physical activity interaction models provided a more detailed examination of whether the observed patterns differed between girls and boys. Together, these methodological features increased the depth and interpretability of the findings and may support the development of more targeted pediatric assessment and prevention strategies.

## 5. Conclusions

Physically inactive children aged 6–9 years showed a less favorable adiposity-related morphological profile than their physically active peers. Specifically, they presented higher body weight, BMI, waist circumference, WHR, WHtR, conicity index, adipose mass, FMI, sum of six skinfolds, and endomorphy, together with lower ectomorphy. These findings indicate that physical activity status was associated with differences in overall and central adiposity-related indicators.

Sex-stratified analyses showed that the differences between physically active and inactive children involved a broader range of adiposity indicators among boys. Age-adjusted interaction models also indicated that several differences, particularly those related to central and total adiposity, were more pronounced in boys than in girls. Overall, this study supports the potential value of complementing BMI with central adiposity indicators, body composition measures, and somatotype analysis when assessing adiposity-related morphological risk in children.

Future longitudinal and multicenter studies using objective physical activity measures, such as accelerometry, and incorporating dietary intake, socioeconomic characteristics, pubertal maturation, and broader cardiometabolic assessments are needed to determine whether these morphological differences persist over time and are consistent across different sociocultural and geographic contexts.

## Figures and Tables

**Table 1:** Anthropometric, body composition, and somatotype indicators according to physical activity status in the total sample.

Variable	Inactive (Mean ± SD) (*n* = 60)	Active (Mean ± SD) (*n* = 66)	*p*-Value	Cohen’s d
Age (years)	7.73 ± 1.19	7.85 ± 1.06	0.566	0.10
Body weight (kg)	36.29 ± 11.50	32.21 ± 7.69	0.020 *	0.42
Height (cm)	130.50 ± 9.79	130.06 ± 9.19	0.794	0.05
BMI (kg/m^2^)	20.78 ± 4.11	18.90 ± 3.05	0.004 *	0.52
Waist circumference (cm)	66.63 ± 9.61	61.50 ± 6.60	0.001 *	0.63
WHR	0.87 ± 0.04	0.84 ± 0.05	<0.001 *	0.68
WHtR	0.51 ± 0.05	0.47 ± 0.05	<0.001 *	0.71
Conicity index	1.18 ± 0.04	1.14 ± 0.04	<0.001 *	0.84
Adipose mass (kg)	13.17 ± 4.83	11.60 ± 3.78	0.044 *	0.36
Muscle mass (kg)	12.58 ± 4.53	10.97 ± 2.90	0.068	0.43
AMR	1.06 ± 0.27	1.09 ± 0.33	0.711	0.07
FMI (kg/m^2^)	7.47 ± 1.86	6.76 ± 1.67	0.025 *	0.41
Sum of skinfolds (mm)	106.78 ± 35.88	91.94 ± 32.85	0.017 *	0.43
Endomorphy	5.93 ± 1.45	5.09 ± 1.70	0.003 *	0.53
Mesomorphy	5.42 ± 1.16	4.93 ± 1.35	0.072	0.39
Ectomorphy	1.17 ± 1.02	1.80 ± 1.38	0.004 *	0.52

Values are presented as mean ± standard deviation. BMI, body mass index; WHR, waist-to-hip ratio; WHtR, waist-to-height ratio; AMR, adipose-to-muscle ratio; FMI, fat mass index; SD, standard deviation. Cohen’s d represents the standardized difference between physically active and inactive children. * *p* < 0.05 was considered statistically significant.

**Table 2:** Anthropometric, body composition, and somatotype indicators according to physical activity status in girls.

Variable	Inactive (Mean ± SD) (*n* = 22)	Active (Mean ± SD) (*n* = 34)	*p*-Value	Cohen’s d
Age (years)	7.45 ± 1.13	7.76 ± 1.15	0.488	0.27
Body weight (kg)	34.71 ± 12.19	32.21 ± 7.69	0.142	0.26
Height (cm)	128.60 ± 10.03	130.66 ± 9.76	0.597	0.21
BMI (kg/m^2^)	20.50 ± 4.91	17.35 ± 3.53	0.008 *	0.76
Waist circumference (cm)	64.59 ± 6.16	60.09 ± 6.11	0.021*	0.73
WHR	0.84 ± 0.03	0.82 ± 0.05	0.036 *	0.46
WHtR	0.50 ± 0.06	0.48 ± 0.06	0.040 *	0.33
Conicity index	1.16 ± 0.03	1.14 ± 0.05	0.047 *	0.46
Adipose mass (kg)	12.34 ± 4.53	12.35 ± 4.16	0.504	0.01
Muscle mass (kg)	12.29 ± 4.98	11.39 ± 3.56	0.546	0.23
AMR	1.04 ± 0.24	1.12 ± 0.33	0.451	0.28
FMI (kg/m^2^)	7.26 ± 1.77	7.10 ± 1.64	0.731	0.09
Sum of skinfolds (mm)	103.64 ± 35.02	99.06 ± 31.76	0.730	0.14
Endomorphy	6.19 ± 1.51	5.52 ± 1.64	0.037 *	0.42
Mesomorphy	5.14 ± 1.44	4.86 ± 1.41	0.612	0.20
Ectomorphy	1.38 ± 1.33	1.72 ± 1.39	0.528	0.25

Values are presented as mean ± standard deviation. BMI, body mass index; WHR, waist-to-hip ratio; WHtR, waist-to-height ratio; AMR, adipose-to-muscle ratio; FMI, fat mass index; SD, standard deviation. Cohen’s d represents the standardized difference between physically inactive and active girls. * *p* < 0.05 was considered statistically significant.

**Table 3:** Anthropometric, body composition, and somatotype indicators according to physical activity status in boys.

Variable	Inactive (Mean ± SD) (*n* = 38)	Active (Mean ± SD) (*n* = 32)	*p*-Value	Cohen’s d
Age (years)	7.89 ± 1.24	7.94 ± 1.00	0.880	0.04
Body weight (kg)	37.21 ± 11.32	30.94 ± 5.69	0.006 *	0.68
Height (cm)	131.61 ± 9.89	129.43 ± 8.98	0.061	0.23
BMI (kg/m^2^)	20.94 ± 3.77	18.43 ± 2.53	0.010 *	0.77
Waist circumference (cm)	67.82 ± 9.49	60.88 ± 4.85	<0.001 *	0.91
WHR	0.89 ± 0.04	0.86 ± 0.03	<0.001 *	0.84
WHtR	0.51 ± 0.05	0.47 ± 0.04	<0.001 *	0.88
Conicity index	1.19 ± 0.04	1.15 ± 0.03	<0.001 *	1.12
Adipose mass (kg)	13.65 ± 5.12	10.80 ± 3.35	0.010 *	0.65
Muscle mass (kg)	12.75 ± 4.45	10.52 ± 2.07	0.239	0.63
AMR	1.08 ± 0.30	1.05 ± 0.35	0.214	0.09
FMI (kg/m^2^)	7.60 ± 1.94	6.39 ± 1.68	0.015 *	0.66
Sum of skinfolds (mm)	108.61 ± 37.67	84.38 ± 33.83	0.003 *	0.67
Endomorphy	5.78 ± 1.46	4.63 ± 1.73	0.012 *	0.72
Mesomorphy	5.57 ± 1.00	5.00 ± 1.36	0.190	0.48
Ectomorphy	1.04 ± 0.81	1.89 ± 1.43	0.165	0.74

Values are presented as mean ± standard deviation. BMI, body mass index; WHR, waist-to-hip ratio; WHtR, waist-to-height ratio; AMR, adipose-to-muscle ratio; FMI, fat mass index; SD, standard deviation. Cohen’s d represents the standardized difference between physically inactive and active boys. * *p* < 0.05 was considered statistically significant.

**Table 4:** Age-adjusted physical activity status × sex interaction models for anthropometric, body composition, and somatotype indicators.

Variable	Interaction β Coefficient	95% CI	*p-*Value
Age (years)	−0.66	−1.40 to 0.08	0.080
Body weight (kg)	−3.13	−6.24 to −0.02	0.048 *
Height (cm)	−5.47	−10.45 to −0.49	0.032 *
BMI (kg/m^2^)	1.59	−0.57 to 3.75	0.145
Waist circumference (cm)	−2.47	−4.84 to −0.10	0.041 *
WHR	−0.03	−0.05 to −0.01	0.018 *
WHtR	−0.02	−0.04 to −0.002	0.026 *
Conicity index	−0.04	−0.07 to −0.01	0.006 *
Adipose mass (kg)	−0.05	−0.09 to −0.01	0.021 *
Muscle mass (kg)	0.00	−0.03 to 0.03	0.884
AMR	−0.13	−0.26 to 0.003	0.058
FMI (kg/m^2^)	−0.02	−0.04 to −0.001	0.047 *
Sum of skinfolds (mm)	−7.51	−14.63 to −0.39	0.039 *
Endomorphy	0.06	−0.37 to 0.49	0.781
Mesomorphy	0.56	−0.10 to 1.22	0.094
Ectomorphy	0.05	−0.53 to 0.63	0.862

Values are presented as unstandardized β coefficients for the physical activity status × sex interaction, with 95% confidence intervals and *p*-values. Separate multiple linear regression models were fitted for each outcome and adjusted for age. Physical activity status was coded as 0 = inactive and 1 = active, whereas sex was coded as 0 = girls and 1 = boys; therefore, physically inactive children and girls were the reference categories. The interaction coefficient indicates whether the difference between physically active and inactive participants differed according to sex. β, unstandardized regression coefficient; CI, confidence interval; BMI, body mass index; WHR, waist-to-hip ratio; WHtR, waist-to-height ratio; AMR, adipose-to-muscle ratio; FMI, fat mass index. * Statistically significant at *p* < 0.05.

## Data Availability

The data that support the findings of this study are available from the corresponding author upon reasonable request.

## Abbreviations

The following abbreviations are used in this manuscript:

AMR	Adipose-to-muscle ratio
BMI	Body mass index
CI	Confidence interval
ES	Effect size
FMI	Fat mass index
ISAK	International Society for the Advancement of Kinanthropometry
PA	Physical activity
PAQ-C	Physical Activity Questionnaire for Older Children
SD	Standard deviation
WHO	World Health Organization
WHR	Waist-to-hip ratio
WHtR	Waist-to-height ratio
